# MDEB-YOLO: A Lightweight Multi-Scale Attention Network for Micro-Defect Detection on Printed Circuit Boards

**DOI:** 10.3390/mi17020192

**Published:** 2026-01-30

**Authors:** Xun Zuo, Ning Zhao, Ke Wang, Jianmin Hu

**Affiliations:** 1 School of Information Engineering, Wuhan University of Technology, Wuhan 430070, China; 2 School of Information Engineering, Wuhan Huaxia Institute of Technology, Wuhan 430223, China; 3 School of Information Engineering, Hubei University of Economics, Wuhan 430205, China; 4 Hubei Key Laboratory of Digital Finance Innovation, Hubei University of Economics, Wuhan 430205, China

**Keywords:** PCB defect detection, MDEB-YOLO, efficient multi-scale deformable attention (EMDA), feature pyramid network, lightweight network

## Abstract

Defect detection on Printed Circuit Boards (PCBs) constitutes a pivotal component of the quality control system in electronics manufacturing. However, owing to the intricate circuitry structures on PCB surfaces and the characteristics of defects—specifically their minute scale, irregular morphology, and susceptibility to background texture interference—existing generic deep learning models frequently fail to achieve an optimal equilibrium between detection accuracy and inference speed. To address these challenges, this study proposes MDEB-YOLO, a lightweight real-time detection network tailored for PCB micro-defects. First, to enhance the model’s perceptual capability regarding subtle geometric variations along conductive line edges, we designed the Efficient Multi-scale Deformable Attention (EMDA) module within the backbone network. By integrating parallel cross-spatial channel learning with deformable offset networks, this module achieves adaptive extraction of irregular concave–convex defect features while effectively suppressing background noise. Second, to mitigate feature loss of micro-defects during multi-scale transformations, a Bidirectional Residual Multi-scale Feature Pyramid Network (BRM-FPN) is proposed. Utilizing bidirectional weighted paths and residual attention mechanisms, this network facilitates the efficient fusion of multi-view features, significantly enhancing the representation of small targets. Finally, the detection head is reconstructed based on grouped convolution strategies to design the Lightweight Grouped Convolution Head (LGC-Head), which substantially reduces parameter volume and computational complexity while maintaining feature discriminability. The validation results on the PKU-Market-PCB dataset demonstrate that MDEB-YOLO achieves a mean Average Precision (mAP) of 95.9%, an inference speed of 80.6 FPS, and a parameter count of merely 7.11 M. Compared to baseline models, the mAP is improved by 1.5%, while inference speed and parameter efficiency are optimized by 26.5% and 24.5%, respectively; notably, detection accuracy for challenging mouse bite and spur defects increased by 3.7% and 4.0%, respectively. The experimental results confirm that the proposed method outperforms state-of-the-art approaches in both detection accuracy and real-time performance, possessing significant value for industrial applications.

## 1. Introduction

With the sustained development of the electronics industry, the Printed Circuit Board (PCB), serving as the core interconnection structure and foundational support platform for integrated circuits, has been extensively applied in mobile phones, edge computing devices, and various intelligent terminals [1]. Driven by the escalating demands for performance, power efficiency, and integration in electronic products, PCB design and manufacturing processes are becoming increasingly complex, evolving towards miniaturization, high-density interconnection, and multi-layer stacking structures [2]. In this context, PCBs are more prone to various structural and process defects during manufacturing and soldering, which may lead to circuit functional failure, compromised system reliability, or even safety hazards [3]. Consequently, implementing rapid and accurate detection of PCB defects during production is imperative for enhancing production line yield, reducing maintenance costs, and ensuring the quality of electronic products [4].

From an industrial application perspective, the detection of irregular surface defects on PCBs has emerged as a representative and challenging problem in the field of object detection. Although PCB images acquired by industrial cameras typically possess high resolution and rich textural details [5], they contain numerous micro-defects that are difficult to distinguish. In particular, defects such as concave–convex micro-notches distributed along the edges of complex conductive lines are characterized by their extremely small spatial scale, concealed local geometric morphology, and blurred structural boundaries. Under a multi-scale background, such defects occupy a negligible proportion of the total area and are easily confused with normal process fluctuations, such as variations in line width or manufacturing tolerances. Furthermore, background interference arising from complex circuit topologies and multi-layer superimposition further weakens the model’s response capability to these minute geometric features, leading to the dual challenge of high false-negative rates and high false-positive rates during the detection process [6]. Finally, deep learning has achieved remarkable success in industrial object detection. However, its widespread adoption in industrial quality control applications has raised growing concerns regarding transparency, explainability, and accountability, and interpreting the decision-making process remains a significant challenge [7].

In recent years, deep-learning-based object detection methods have been widely applied to PCB defect inspection, including two-stage detectors such as Faster R-CNN [8] and single-stage detectors such as RetinaNet [9], the YOLO family [10], and the transformer-based RT-DETR [11]. However, during multi-scale feature fusion, small objects are prone to information loss in the top–down propagation process, resulting in degraded multi-scale feature representations with blurred boundaries [12]. Moreover, these models generally lack explicit feature enhancement mechanisms tailored to concave–convex micro-notch defects on PCBs. To improve multi-scale feature fusion, many studies have focused on enhancing feature representation capability. Libra R-CNN [13] balances semantic information by integrating features at the same depth to strengthen multi-level representations, AugFPN [14] injects diverse spatial contextual information from high-level feature maps into the top–down pathway through residual branches, and CARAFE [15] reassembles features at each spatial location via weighted combinations to aggregate information within a large receptive field. Although the design paradigm of feature enhancement followed by multi-scale feature fusion has been demonstrated to improve task-specific feature representations in object detection, existing general-purpose detection frameworks still exhibit notable limitations in modeling concave–convex micro-notch defects on PCBs. Specifically, they lack explicit modeling of defect topological structures and local displacement relationships, which constrains their ability to represent fine-grained concave–convex contours, and they also struggle to achieve an effective balance between detection accuracy and inference efficiency.

Addressing the aforementioned issues, this paper focuses on the detection of concave–convex micro-notch defects along conductive line edges in real-world PCB manufacturing scenarios. From the perspective of “Enhance–Fuse–Align,” we re-examine the detection pipeline and propose MDEB-YOLO, a lightweight real-time detection model specifically designed for micro-defect inspection in PCBs. The objective is to achieve an optimal balance among detection accuracy, model complexity, and inference speed. The primary work and contributions of this paper are summarized as follows:

(1) Construction of the Efficient Multi-scale Deformable Attention module to significantly enhance the feature representation of micro-geometric structures. Addressing the problem that concave–convex micro-defects on conductive lines have minute target dimensions, subtle local morphologies, and are difficult for models to focus on, the EMDA module utilizes cross-spatial channel re-weighting and deformable offset modeling. While maintaining the continuity constraints of the overall circuit structure, it adaptively perceives pixel-level shifts and local concave–convex deformations at the edges of conductive lines, thereby resolving the inability of conventional convolutions to capture irregular edge features.
(2) Design of the Bidirectional Residual Multi-scale Feature Pyramid Network to improve robust detection capabilities under complex backgrounds. Addressing the issues of extremely low defect area ratios and susceptibility to multi-scale background interference, this network constructs bidirectional feature weighting paths operating in both top–down and bottom–up directions. By effectively suppressing redundant channel features, the BRM-FPN leverages residual connections and multi-scale information interaction to balance global semantic information with local textural details, ensuring significant responses for micro-defects across different feature hierarchies.
(3) Proposal of the Lightweight Grouped Convolution Detection Head to facilitate industrial-grade real-time inference and efficient deployment. To solve the problems of large parameter redundancy and high inference latency in existing detection networks, this study reconstructs the traditional YOLO detection head based on grouped convolution concepts. The LGC-Head employs an optimized grouping strategy that allows convolution kernels to operate only on specific channel subsets. This significantly reduces parameter scale and computational complexity while preserving critical feature discriminability, thereby substantially improving the model’s inference efficiency and adaptability for deployment on resource-constrained industrial devices.

On this basis, a systematic evaluation of the MDEB-YOLO model’s detection performance, model complexity, and inference speed was conducted on a public PCB defect dataset. Comparative analysis with various typical object detection methods validates the effectiveness and superiority of the proposed method in the task of detecting concave–convex micro-defects.

## 2. Related Work

### 2.1. Traditional PCB Inspection Methodologies

Early quality control protocols for PCBs predominantly relied upon manual visual inspection [16]. Inspectors typically employed magnifying lenses or microscopes to scrutinize the PCB surface point-by-point to identify defects. Although this approach offered a degree of flexibility for small-batch production, its efficiency was inherently low, and the results were highly susceptible to the inspector’s experience level, visual fatigue, and subjective judgment fluctuations [17]. Confronted with micro-defects characterized by minute dimensions or concealed morphologies, manual inspection frequently failed to guarantee stability, rendering it inadequate for the rigorous efficiency and consistency standards of modern large-scale electronics manufacturing [18]. With the advancement of production line automation, automated inspection technologies have gradually superseded manual inspection, becoming the primary means of PCB quality control.

Automated Optical Inspection (AOI) constitutes a paradigmatic solution integrating machine vision, photoelectric sensing, and pattern recognition technologies to automatically identify, localize, and classify surface defects. It remains the most widely applied technique in traditional industrial production [19]. AOI systems acquire PCB images via multi-angle, multi-source high-resolution imaging systems and, combining image segmentation with feature extraction algorithms, compare extracted features against Golden Templates or design data to complete large-scale surface defect screening within short durations. However, in complex practical production environments, factors such as illumination fluctuations, high specular reflection interference, and circuit structural complexity significantly attenuate the robustness of AOI systems. Particularly when addressing micro-defects, such as subtle edge concavities and convexities, these methods exhibit marked limitations, frequently precipitating both false positives and false negatives [20].

### 2.2. Machine-Learning-Based PCB Detection

With the evolution of artificial intelligence, machine learning paradigms have gained extensive traction in the fields of image analysis and surface defect detection. Machine-learning-based PCB defect detection approaches typically adopt supervised representation learning strategies, formulating the detection problem as classification tasks at varying granularities, including coarse-grained discrimination at the image level, defect recognition at the local region level, and fine-grained segmentation at the pixel level [21]. For instance, Yun et al. [22] employed a Support Vector Machine (SVM) classifier combined with a hierarchical circular illumination scheme to inspect PCB solder joints, effectively distinguishing between over-soldered and well-soldered samples. Belbachir et al. [23] constructed a PCB defect detection system based on Wavelet Transform (WVT) [24] and Multi-Layer Perceptron (MLP) neural networks [25], achieving automatic recognition of multiple defect categories through the synergy of frequency-domain features and non-linear classifiers.

Notwithstanding the improvements in detection automation, machine-learning-based schemes remain insufficient for micro-defect detection tasks on high-density PCBs. On one hand, such methods frequently rely on hand-crafted features or specific patching strategies, making classifier performance highly contingent upon the quality of feature engineering; when defect morphology, scale, or imaging conditions vary, the feature engineering workflow often requires redesign or adjustment. On the other hand, regarding ultra-small targets or subtle concave–convex structural defects, the feature expression capability and spatial resolution of traditional machine learning models remain limited, resulting in detection accuracy and generalization capabilities that fail to meet the demands of complex industrial scenarios.

### 2.3. Deep-Learning-Based PCB Detection

The burgeoning development of deep learning has significantly propelled innovations in PCB defect detection technology, enabling detection networks to output target localization and category information end-to-end within a unified framework. Hu et al. [26] proposed a method combining an improved Faster R-CNN with a Feature Pyramid Network (FPN), utilizing ResNet50 as the feature extraction backbone and leveraging the pyramid structure to fuse shallow details with deep semantics, thereby enhancing detection accuracy for small-scale defects. Wu et al. [27], based on transfer learning principles, combined ResNet101 with Mask R-CNN for solder joint detection, demonstrating superior generalization capabilities across multiple soldering defect categories. Although such two-stage detectors possess advantages in detection accuracy, their complex network architectures, substantial parameter volume, and high inference overhead render them difficult to satisfy the stringent real-time requirements of high-speed production lines.

To alleviate computational pressure and accelerate processing speed, researchers have initiated the incorporation of lightweight backbone networks into industrial vision tasks. Representative examples include the MobileNet series (V1–V3) [28,29,30], GhostNet [31], the ShuffleNet series [32,33], and EfficientNet [34]. These networks employ strategies such as depth-wise separable convolutions, feature reuse, and synergistic searches for width and depth, achieving faster inference speeds with lower parameter counts and computational loads. Embedding such lightweight networks into defect detection or classification frameworks facilitates model deployment on resource-constrained industrial devices. However, in micro-defect detection tasks, excessive feature compression often undermines the ability to characterize fine-grained geometric structures, resulting in decreased detection accuracy and elevated false-negative rates for small targets.

With the rise of Vision Transformer (ViT) models, several studies have attempted to introduce self-attention mechanisms into PCB defect detection. An et al. [35] proposed the LPViT model, enhancing attention to detailed regions while maintaining high processing speeds; this model improves training robustness through label smoothing strategies and utilizes mask block prediction mechanisms to promote sufficient learning of deep features. Liu et al. [36] proposed the RDetr model, combining Deformable Transformers with a pyramidal feature fusion structure for PCB micro-defect detection. RDetr introduces a Multi-scale Feature Enhancement module (MNT) to intensify small defect features and suppress background interference, employs an MPFF module to mitigate the loss of small defect features during network deepening, and implements adaptive detection of defect regions via an ADD module [37]. These methods have achieved a certain balance between detection accuracy and inference speed, validating the efficacy of attention mechanisms and Transformer architectures in PCB defect detection.

### 2.4. XAI in Industrial Inspection

In industrial inspection, the decisions made by models directly impact product quality, production safety, and economic outcomes. However, most deep learning detectors remain black boxes, making it difficult for engineers to understand why defects are detected or missed. Explainable AI (XAI) provides intuitive explanations for model predictions by revealing the contributions of key visual features. Existing methods mainly include gradient/activation mapping, perturbation analysis, and attention-based explanations. Gradient or activation mapping methods, such as Grad-CAM [38], generate attention heatmaps using internal gradients to visually highlight important regions of the model. Perturbation methods, such as RISE [39], assess pixel contributions by randomly occluding image regions, without the need to access the internal structure of the model. With the development of vision transformers, attention-based explanation methods [40] have emerged, which can trace information flow across layers and enable global semantic explanations.

However, aligning explainability metrics with human cognitive expectations remains a key challenge for XAI in industrial applications. To further enhance reliability in industrial scenarios, physically explainable AI has emerged. Unlike conventional XAI that primarily focuses on feature attention, physically explainable AI emphasizes that model decisions should conform to real-world physical laws or structural constraints. For example, Huang et al. [41] proposed integrating physical priors into convolutional networks to explain predictions, ensuring consistency between model decisions and the physical characteristics of SAR images; Cuomo et al. [42] introduced physics-based constraints into neural networks to improve generalization and physical consistency. In the context of industrial inspection, physical priors can guide models to identify complex defects, enhancing the accuracy and reliability of microstructural defect detection while also increasing engineers’ trust in the model predictions.

In summary, although existing deep-learning-based PCB defect detection methods have achieved remarkable progress in terms of overall detection performance and real-time efficiency, they generally lack targeted structural modeling and feature enhancement mechanisms for the specific class of defects characterized by concave–convex micro-notches along conductive line edges. Such defects are morphologically subtle, occupy extremely small area proportions, and are highly susceptible to interference from complex backgrounds. Most existing approaches tend to treat these defects as conventional detection targets, without sufficiently exploiting circuit continuity, local geometric deformation characteristics, or fine-grained topological relationships. As a result, model decisions rely heavily on implicit feature representations and lack explicit structural semantic constraints. Consequently, in practical inspection scenarios, the detection of such micro-structural defects still suffers from high false-negative rates and elevated false-positive rates. Moreover, existing methods provide limited support for explainability, making it difficult to clearly reveal the key regions attended by the model and their correspondence to actual defect morphologies. This hinders effective result verification and failure diagnosis by engineers, thereby further restricting the deployment and application of these methods in high-reliability industrial inspection scenarios.

## 3. Methodology

### 3.1. MDEB-YOLO Network

**Figure 1:**
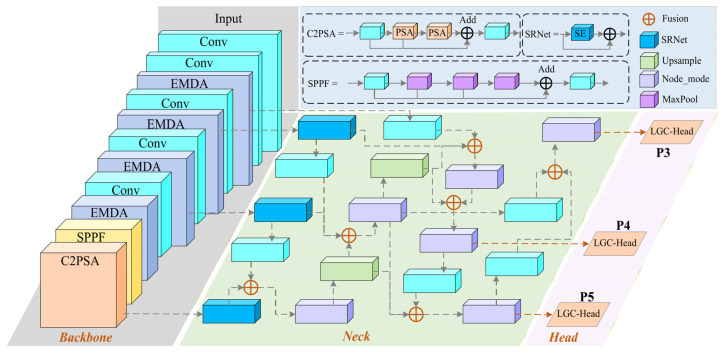
The overall architecture of the MDEB-YOLO network. Input PCB images first enter the backbone network, sequentially passing through standard convolution layers and EMDA modules to complete progressive downsampling and feature extraction; in the deep layers, multi-scale spatial aggregation is performed via SPPF, followed by further enhancement in deep semantic features via the C2PSA structure, yielding multi-scale backbone feature maps P3, P4, and P5. Subsequently, these three feature streams are fed into the BRM-FPN neck network, where they undergo SRNet residual attention enhancement, upsampling/downsampling fusion, and C3k2_faster refinement to form enhanced features $F_{3}^{f}$, $F_{4}^{f}$ and $F_{5}^{f}$. Finally, the three enhanced feature streams are input into the corresponding LGC-Head detection heads, outputting bounding box and category predictions at P3, P4, and P5 to achieve joint detection of PCB defects across different scales.

The MDEB-YOLO consists of a backbone feature extraction network, a BRM-FPN neck for feature fusion, and an LGC-Head lightweight detection head. Different from the traditional YOLO backbone architecture, MDEB-YOLO introduces a feature enhancement module at the backbone feature extraction stage and is designed under a unified “feature enhancement–feature fusion–feature alignment” framework. This structure effectively strengthens the modeling capability of fine-grained structural information along PCB trace edges, thereby improving the model’s sensitivity to and localization accuracy of tiny defect feature. The overall framework is illustrated in Figure 1. Input PCB images are initially processed by the backbone network, traversing multiple layers of standard convolutions and EMDA modules to progressively achieve spatial downsampling and feature extraction. This yields multi-scale feature maps encapsulating both fine-grained geometric structures and global semantic information. In the deep stages of the backbone, Spatial Pyramid Pooling—Fast (SPPF) and C2PSA structures are incorporated to expand the effective receptive field and enhance context encoding capabilities. This enables the network to more adequately characterize directional changes in conductive lines and their local concave–convex notch morphologies, providing semantically rich high-level features for subsequent multi-scale fusion.

Commencing with raw resolution input, the backbone network alternates between stacking standard convolutional layers and EMDA modules to form three downsampling stages, corresponding to three feature layers of varying resolutions: P3, P4, and P5. The EMDA module performs directional modeling and response enhancement for concave–convex micro-notch structures along conductive line edges across all scales. This ensures that minute geometric anomalies, such as mouse bites and spurs, acquire significantly elevated response intensity and distinctiveness within the feature space. The deepest features undergo spatial aggregation across multiple receptive field scales via SPPF, followed by the reinforcement of inter-channel dependencies and long-range contextual information via C2PSA. This process effectively suppresses background interference unrelated to defects while maintaining the integrity of the overall structure.

The backbone-output feature layers P3, P4, and P5 are subsequently fed into the BRM-FPN neck network, where bidirectional, multi-level, and multi-source feature fusion is executed. BRM-FPN first utilizes the embedded SRNet to perform residual attention enhancement and channel alignment on multi-scale features, yielding initial features F3, F4, and F5. Subsequently, the network alternates between bottom–up and top–down directions, executing upsampling, downsampling, and C3k2_faster refinement operations. Through repeated cross-layer fusion, the enhanced features $F_{3}^{f}$, $F_{4}^{f}$, and $F_{5}^{f}$ are formed. This process fully introduces deep semantic information while preserving shallow edge textures, ensuring that concave–convex micro-defects possess more concentrated and highly discriminative feature representations across different spatial scales.

In the detection phase, the enhanced features $F_{3}^{f}$, $F_{4}^{f}$, and $F_{5}^{f}$ serve as inputs to the LGC-Head lightweight grouped detection head. LGC-Head constructs bounding box regression branches and category prediction branches separately at three scales. By employing a grouped convolution design to implement grouping operations for convolution kernels and channels, it retains necessary cross-channel interaction capabilities while significantly reducing parameter volume and computational load. The network ultimately completes multi-scale detection of various PCB defects—including missing holes, mouse bites, open circuits, shorts, spurs, and spurious copper—jointly across the P3, P4, and P5 output layers, providing efficient and stable prediction results for industrial online inspection.

### 3.2. EMDA Module

In the detection task of concave–convex micro-notch defects along conductive line edges, defects often occupy minimal pixels, exhibit subtle morphological changes, and are situated adjacent to complex circuitry backgrounds. Conventional convolutions extract local textural features at fixed sampling positions; consequently, their perceptual capability regarding structures that simultaneously rely on precise edge positioning and continuous geometric deformation is limited. This often leads to feature responses being submerged by the background or a lack of focused attention on defect locations. To explicitly strengthen the modeling capability for conductive line geometric contours and local concave–convex morphologies—without altering the overall backbone structure—it is imperative to introduce an enhanced convolution unit capable of simultaneously attending to spatial positional information and deformable sampling relationships during the feature extraction stage.

**Figure 2:**
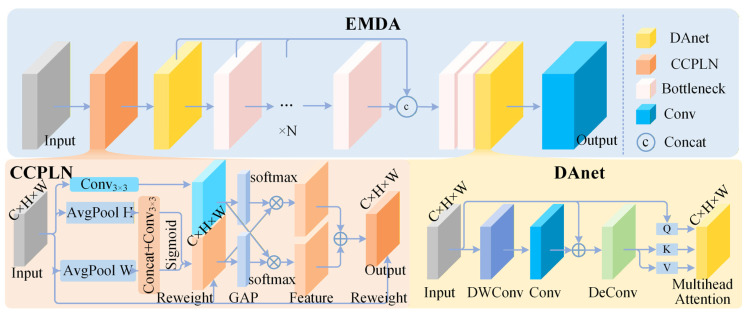
Schematic diagram of the EMDA convolution module structure. Input features are first divided into several sub-feature groups along the channel dimension, each of which is fed into the CCPLN module for parallel cross-spatial channel learning. CCPLN generates position-dependent attention weights via 1D global average pooling along horizontal and vertical directions combined with 1 × 1 convolutions while simultaneously acquiring local spatial context information via a 3 × 3 convolution branch. Weighted features from both branches are fused after global re-calibration to yield the enhanced feature map. This feature map is then input into the DAnet module, where query, key, and value are generated via depth-wise separable convolutions and deconvolutions. Sampling offsets are obtained from the offset prediction sub-network, followed by multi-head attention calculation after bilinear interpolation sampling at offset positions. Finally, the multi-head attention output is fused with the input feature via residual connection to obtain the EMDA output feature focused on the concave–convex micro-notch structures of conductive line edges.

Based on these considerations, this paper designs the EMDA convolution module to perform directional reinforcement of multi-scale features within the backbone network. This module comprises two components: a Parallel Cross-Spatial Channel Learning Network (CCPLN) and a Deformable Attention sub-network (DAnet). The former adopts a dual-branch architecture. The 1 × 1 convolution branch jointly models channel grouping and orthogonal-direction positional encoding [38], while the 3 × 3 convolution branch aggregates multi-scale spatial structural information, enabling adaptive reweighting of local edge regions and long-range contextual information. The latter proactively selects key positions spatially related to defects by predicting sampling offsets and executing multi-head attention calculations at deformed positions, thereby realizing fine-grained characterization of minute geometric structures. Input features first undergo channel and spatial dimension recalibration within the CCPLN, followed by offset sampling and attention aggregation in the DAnet. Finally, the enhanced feature map is output via a residual connection. The overall structure is illustrated in Figure 2.

Let the input feature map be $X\in R^{C\times H\times W}$. EMDA first uniformly divides it into G sub-feature groups along the channel dimension, yielding the following:

(1) $$X=\left[X_{0},X_{1},\ldots ,X_{G-1}\right],\;X_{i}\in R^{\left(C/G\right)\times H\times W}$$

In Equation (1), the input feature map is uniformly partitioned along the channel dimension into G subgroups. To strike an optimal balance between computational efficiency and channel representation diversity, we set the number of groups as G=C/16. This configuration was determined through a grid search over G∈{C/8,C/16,C/32,C/64,C/128} on the validation set: when G is too small, interchannel interaction is limited, hindering the modeling of complex edge structures; conversely, when G is too large, computational overhead increases significantly while performance gains saturate. The experimental results show that G=C/16 effectively preserves channel diversity while reducing per-group computational cost, thereby providing a solid foundation for subsequent attention computations. Each sub-feature group is independently fed into the CCPLN for joint cross-spatial channel modeling.

CCPLN enhances the response to precise geometric coordinates of wire edges by explicitly modeling their orthogonal position sensitivity through 1D global pooling in both horizontal and vertical directions. Within the CCPLN, to simultaneously encode positional information in both horizontal and vertical directions, 1D global average pooling is applied to each sub-feature $X_{i}$ to obtain two 1D descriptor vectors. The pooling result along the vertical direction is denoted as $z_{c}^{H}\left(h\right)$, and along the horizontal direction as $z_{c}^{W}\left(w\right)$, calculated as follows:

(2) $$z_{c}^{H}\left(h\right)=\frac{1}{W}\sum_{w=0}^{W-1}x_{c}\left(h,w\right),\;z_{c}^{W}\left(w\right)=\frac{1}{H}\sum_{h=0}^{H-1}x_{c}\left(h,w\right)$$

where $x_{c}\left(h,w\right)$ is the feature response of the c-th channel at position $\left(h,w\right)$. The pooling vectors from both directions are concatenated along the spatial dimension and input into a 1 × 1 convolution layer without channel dimensionality reduction. The convolution output is then split into two 1D vectors, which pass through a Sigmoid activation to yield the vertical position attention map $A_{H}\in R^{C\times H\times 1}$ and the horizontal position attention map $A_{W}\in R^{C\times 1\times W}$. These are combined via an outer product operation to obtain the attention weights for the 1 × 1 branch:

(3) $$A_{1\times 1}=A_{H}⊗A_{W}$$

where ⊗ denotes the outer product operation. This branch emphasizes precise positional information of conductive line edges along horizontal and vertical axes.

To supplement local spatial context information, CCPLN introduces a 3 × 3 convolution branch to perceive neighborhood structures at a larger scale. A convolution operation with a 3 × 3 kernel size and consistent input–output channel counts is applied to each sub-feature $X_{i}$, followed by a Sigmoid function to obtain spatial attention weights:

(4) $$A_{3\times 3}=\sigma \left({\mathrm{Conv}}_{3\times 3}\left(X_{i}\right)\right)$$

where σ(⋅) denotes Sigmoid activation. The 3 × 3 branch focuses more on structural continuity and background context within local regions. Subsequently, the attention weights obtained from both branches are element-wise multiplied with the original sub-features to obtain weighted features:

(5) $$F_{i}^{1\times 1}=X_{i}⊙A_{1\times 1},\;F_{i}^{3\times 3}=X_{i}⊙A_{3\times 3}$$

where ⊙ denotes element-wise multiplication. The former reinforces responses in position-sensitive regions of conductive line edges, while the latter reinforces long-range context information related to defects. Global pooling and normalization are applied to $F_{i}^{1\times 1}$ and $F_{i}^{3\times 3}$ to obtain global importance weights for both branches, which are then multiplied channel-wise with the corresponding features and fused via addition to form the output feature $X_{\mathrm{cc}}\in R^{C\times H\times W}$ after parallel cross-spatial channel learning. This process realizes joint modeling of local and global structural variations, enabling the network to respond more sensitively to geometric changes near concave–convex micro-notches.

Upon obtaining the enhanced feature $X_{\mathrm{cc}}$, EMDA further introduces DAnet to achieve adaptive sampling by predicting spatial offsets and applies a multi-head attention mechanism at the deformed positions to capture fine-grained structural features of irregular defects such as concave–convex micro-notches. Let the input feature to DAnet be $x\in R^{H\times W\times C}$. First, a uniform grid point set $p\in R^{H_{G}\times W_{G}\times 2}$ is constructed on the feature plane as reference sampling positions. The query vector q is obtained via linear projection:

(6) $$q=xW_{q}$$

And it is fed into the lightweight offset prediction sub-network $\theta_{\mathrm{offset}}\left(\cdot \right)$ to generate offsets Δp for each reference point:

(7) $$\Delta p=\theta_{\mathrm{offset}}\left(q\right)$$

Simultaneously, linear mapping is applied to the input feature to obtain the key vector $k=xW_{k}$ and value vector $v=xW_{v}$. Bilinear interpolation sampling is performed at the offset position p+Δp to obtain the deformed feature:

(8) $$\tilde{x}=\phi \left(x;p+\Delta p\right)$$

where ϕ(⋅) is the sampling function. On this basis, DAnet executes multi-head attention calculations on query q, key k, and value v, introducing relative position bias to enhance modeling capabilities for spatial structures. For the m-th attention head, the similarity matrix with position bias is calculated as follows:

(9) $$\alpha^{\left(m\right)}=\mathrm{softmax}\left(\frac{q^{\left(m\right)}{\left(k^{\left(m\right)}\right)}^{⊤}}{\sqrt{d}}+B^{\left(m\right)}\right)$$

where d is the scaling factor for the channel dimension, and $B^{\left(m\right)}$ is the bias matrix generated by the relative position embedding function $\phi \left(R;p+\Delta p\right)$. The attention weights are then used to perform a weighted sum of the value vectors, yielding the output of that attention head:

(10) $$z^{\left(m\right)}=\alpha^{\left(m\right)}v^{\left(m\right)}$$

Outputs from all attention heads are concatenated along the channel dimension and passed through a linear mapping. Finally, a residual connection with the input feature is employed to obtain the final output feature of the EMDA convolution module.

Through the aforementioned two-stage modeling process, CCPLN achieves explicit positional encoding along horizontal and vertical directions and local context aggregation based on channel grouping, while DAnet selectively focuses on key positions near conductive line edges spatially via deformable sampling and multi-head attention. The combination of the two enables EMDA to adaptively focus on micro-geometric structures such as concave–convex micro-notches within complex backgrounds, providing more discriminative feature representations for subsequent feature fusion and detection.

### 3.3. BRM-FPN Neck Feature Fusion Network

**Figure 3:**
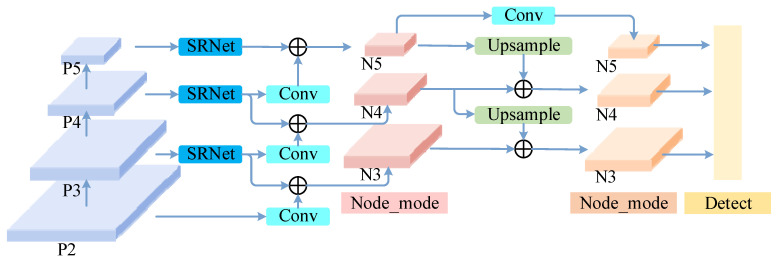
Schematic diagram of the BRM-FPN neck feature fusion network structure. The left side shows multi-scale features $P_{2}$, $P_{3}$, $P_{4}$, and $P_{5}$ from the backbone network. $P_{3}$, $P_{4}$, and $P_{5}$ are sequentially fed into SRNet for residual attention enhancement and channel alignment to obtain initial features $F_{3}$, $F_{4}$, and $F_{5}$; $P_{2}$ forms the auxiliary high-resolution feature $R_{3}$ via convolution and C3k2_faster refinement. The middle section shows the bottom–up path: $F_{4}$ is downsampled and fused with $F_{5}$, then processed by C3k2_faster to output $R_{5}$; $R_{5}$ is upsampled and fused with the downsampled $F_{3}$ and $F_{4}$ to obtain $R_{4}$; subsequently, $R_{4}$ is upsampled and refined multiple times with $F_{3}$ and its downsampled features to obtain the high-resolution enhanced feature $F_{3}^{f}$. The right side shows the top–down path: $R_{3}$ and $F_{3}^{f}$ are downsampled and undergo four-source fusion with $R_{4}$ and the upsampled $R_{5}$ to obtain $F_{4}^{f}$; $R_{4}$ and $F_{4}^{f}$ are downsampled and fused with $R_{5}$ to obtain $F_{5}^{f}$. Finally, the multi-scale enhanced features $F_{3}^{f}$, $F_{4}^{f}$, and $F_{5}^{f}$ are sent to the detection head as input to realize joint detection of PCB defects at different scales.

In multi-scale object detection tasks, the neck network assumes a critical role in bridging backbone feature extraction and the detection head, necessitating the preservation of shallow detail information while introducing deep semantic representations to provide compatible feature support for both small and large targets. Addressing defects such as concave–convex micro-notches on PCB conductive lines—which are small in size, subtle in morphology, and easily submerged by the background—this work designs a Bidirectional Residual Multi-scale Feature Pyramid Network (BRM-FPN). This structure explicitly suppresses redundant channel responses during feature fusion, performs multi-scale information interaction via bidirectional paths (bottom–up and top–down), and cooperates with lightweight refinement modules to achieve efficient feature enhancement, providing the detection head with more compact and discriminative multi-scale representations. The specific structure of BRM-FPN is shown in Figure 3.

BRM-FPN receives three feature layers of different scales from the backbone network:

(11) $$P_{3}\in R^{H/8\times W/8\times C},\;P_{4}\in R^{H/16\times W/16\times C},\;P_{5}\in R^{H/32\times W/32\times C}$$

It simultaneously introduces an auxiliary high-resolution branch from the shallower feature layer P2 to supplement edge textures and detailed information. Before entering multi-scale fusion, *F*($P_{3}$,$P_{4}$,$P_{5}$) are individually processed by the Residual Attention Network (SRNet) to suppress redundant channel responses and unify channel counts. SRNet is a channel attention mechanism that suppresses redundant channel responses and unifies the channel dimensions by exploiting global contextual information to recalibrate feature responses. Building upon this, EMDA introduces a learnable residual connection between the original features and the SE-enhanced features, which can be formally expressed as follows:

(12) $$SRNet(P_{i})=P_{i}+F_{SE}(P_{i})$$

where $P_{i}$ denotes the input feature map and $F_{SE}(\cdot )$ represents the SE transformation. The resulting initial multi-scale features are denoted as follows:

(13) $$\begin{array}{c} F_{3}={\mathrm{Conv}}_{1\times 1}\left(P_{3}+\mathrm{SRNet}\left(P_{3}\right)\right)\\ F_{4}={\mathrm{Conv}}_{1\times 1}\left(P_{4}+\mathrm{SRNet}\left(P_{4}\right)\right)\\ F_{5}={\mathrm{Conv}}_{1\times 1}\left(P_{5}+\mathrm{SRNet}\left(P_{5}\right)\right) \end{array}$$

where ${\mathrm{Conv}}_{1\times 1}$ is responsible for channel compression and alignment, and SRNet highlights significant channel responses related to defects through a residual attention structure. The shallow feature P2 first passes through a convolution layer and a C3k2_faster lightweight refinement module to obtain the auxiliary feature $R_{3}$, which is primarily used to supplement high-resolution geometric details in the subsequent fusion stage.

On this basis, BRM-FPN first performs bottom–up fusion to aggregate deep semantic information. The bottom–up path downsamples the medium-scale feature $F_{4}$ to the same resolution as $F_{5}$, aggregates multi-source inputs via the BiFPN-like adaptive weighted fusion operator Fusion, and then outputs the deep semantic feature $R_{5}$ via the lightweight refinement unit Ψ:

(14) $$R_{5}=\Psi \left(Fusion\left(F_{4},F_{5}\right)\right)$$

where Fusion denotes the adaptive weighted fusion of features from different sources, and $\Psi \left(\cdot \right)$ represents the non-linear refinement function based on C3k2_faster.

Subsequently, $R_{5}$ is upsampled to the $P_{4}$ resolution, and $F_{3}$ is simultaneously downsampled to the same resolution. These are input into the Fusion module together with the original medium-scale feature $F_{4}$ to yield the intermediate feature:

(15) $$R_{4}=\Psi \left(Fusion\left(R_{5}↑,F_{3}↓,F_{4}\right)\right)$$

where ↑ and ↓ denote bilinear upsampling and stride-2 downsampling operations, respectively. Next, $R_{4}$ is upsampled to the highest resolution, fused with the original $F_{3}$ and the auxiliary feature obtained by downsampling $P_{2}$, and refined twice consecutively via C3k2_faster to obtain the high-resolution enhanced feature:

(16) $$F_{3}^{f}=\Psi \left(\Psi \left(Fusion\left(R_{4}↑,F_{3},P_{2}↓\right)\right)\right)$$

This stage fully introduces bottom–up high-level semantic information while preserving shallow edge textures, ensuring that fine-grained defects possess clearer and more concentrated responses in high-resolution feature maps.

After aggregating deep semantic information, BRM-FPN then performs top–down fusion to supplement shallow-level details. BRM-FPN downsamples the auxiliary feature $R_{3}$ and the enhanced feature $F_{3}^{f}$ to medium resolution, fuses them with the intermediate feature $R_{4}$ and the feature upsampled from $R_{5}$ via four-source fusion, and refines them to form the final medium-scale fused feature:

(17) $$F_{4}^{f}=\Psi \left(Fusion\left(R_{3}↓,F_{3}^{f}↓,R_{4},R_{5}↑\right)\right)$$

Finally, $R_{4}$ and $F_{4}^{f}$ are downsampled to the minimum resolution and fused with $R_{5}$ to obtain the deep final feature:

(18) $$F_{5}^{f}=\Psi \left(Fusion\left(R_{4}↓,F_{4}^{f}↓,R_{5}\right)\right)$$

Thus, fused features $\left(F_{3}^{f},F_{4}^{f},F_{5}^{f}\right)$ are obtained at three scales, providing multi-scale inputs possessing both global semantics and local details for the subsequent detection head.

During feature fusion, BRM-FPN introduces adaptive weights for each input feature to balance contributions from different levels. Let the input features of a fusion node be $F_{1},F_{2},\ldots ,F_{n}$ and the corresponding non-negative weights be $w_{1},w_{2},\ldots ,w_{n}$; the fusion output can be expressed as follows:

(19) $$F_{\mathrm{out}}=\sum_{i=1}^{n}w_{i}F_{i},\;\sum_{i=1}^{n}w_{i}=1$$

Weights are learned and automatically adjusted during training, thereby dynamically allocating the importance of features at each scale according to defect type and background complexity. By repeating the pattern of weighted fusion and C3k2_faster refinement at each scale, BRM-FPN ensures that the final feature maps undergo at least two non-linear enhancements, integrating high-level semantic information, low-level detail information, and same-level historical states, significantly improving feature discriminability and expression capability for micro-defects.

### 3.4. LGC-Head Lightweight Grouped Detection Head

**Figure 4:**
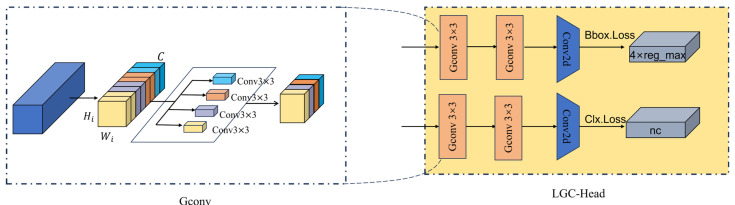
Schematic diagram of the LGC-Head lightweight grouped detection head structure. The left side shows multi-scale enhanced feature maps from the neck network; at each scale, they first enter a Stem module composed of two layers of 3 × 3 grouped convolutions to complete local feature refinement and cross-channel interaction. The middle section shows features at three scales being fed into regression and classification tracks within their respective branches; each track maps the channel count to the target dimension via a single convolution layer. The right side displays the final output, where the regression branch generates a bounding box distribution of dimension 4×reg_max for calculating bounding box loss, and the classification branch generates predictions of dimension $n_{c}$ (number of categories) for calculating classification loss. Multi-scale bounding box and category predictions jointly constitute the detection head output, realizing joint detection of PCB defects at different scales.

In multi-scale detection frameworks, the detection head directly determines the precision and efficiency of bounding box regression and class prediction. Traditional detection heads typically rely on standard convolutions or fully channel-decoupled depth-wise separable convolutions. The former incurs massive computational overhead in high-channel scenarios, hindering industrial online deployment; the latter, while significantly reducing computation, weakens the capability to model fine-grained features by completely severing inter-channel associations—a drawback particularly detrimental to PCB micro-defect detection. To reduce computational costs while maintaining sufficient cross-channel interaction, this paper designs a lightweight detection structure based on grouped convolutions, designated as LGC-Head (Lightweight Grouped Convolution Detection Head). The overall structure is illustrated in Figure 4.

LGC-Head receives enhanced features from the neck network at three scales:

(20) $$F_{i}^{f}\in R^{H_{i}\times W_{i}\times C_{i}},\;i\in 3,4,5$$

It constructs two parallel tracks at each scale: one for bounding box regression and one for category prediction. Both tracks share a consistent structure, comprising a Stem module and a single convolution layer, ultimately outputting the bounding box prediction and category prediction for that scale, respectively. The mapping relationship of the entire detection head can be expressed as follows:

(21) $$LGC-Head\left(F_{3}^{f},F_{4}^{f},F_{5}^{f}\right)\to \{{\mathrm{BBox}}_{P_{3}},{\mathrm{BBox}}_{P_{4}},{\mathrm{BBox}}_{P_{5}}Class_{P_{3}},{\mathrm{Class}}_{P_{4}},{\mathrm{Class}}_{P_{5}}\}$$

where ${\mathrm{BBox}}_{P_{i}}$ represents the bounding box prediction at scale $P_{i}$, and $Class_{P_{i}}$ represents the corresponding category prediction.

At each scale, input features first pass through a Stem module to complete local feature refinement and basic cross-channel interaction. The Stem module consists of two sequentially stacked 3 × 3 grouped convolution layers, with the number of groups adaptively set to $G=C_{i}/16$. For features at scale i, this can be written as follows:

(22) $$Stem_{i}\left(F_{i}^{f}\right)=Conv_{3\times 3}^{G=C_{i}/16}Conv_{3\times 3}^{G=C_{i}/16}F_{i}^{f}$$

where ${\mathrm{Conv}}_{3\times 3}^{\left(G\right)}$ denotes a 3 × 3 convolution operation with G groups. The Stem output is subsequently fed into the regression and classification branches, each completing channel mapping via a single convolution layer. The regression branch outputs 4×reg_max channels, employing a discrete distribution form to represent the four coordinates of the bounding box; the classification branch outputs nc channels, corresponding to the number of defect categories. Consequently, LGC-Head significantly reduces the parameters and computational load of the detection head while ensuring the expressive capability required for detection accuracy.

Analyzing from the perspective of computational complexity, let the input feature channel count be $C_{\mathrm{in}}$, the output channel count be $C_{\mathrm{out}}$, the kernel size be *K* × *K*, and the feature map spatial dimensions be *H* × *W*. The computational load for standard convolution is as follows:

(23) $${\mathrm{FLOPs}}_{\mathrm{conv}}=C_{\mathrm{in}}\times C_{\mathrm{out}}\times K\times K\times H\times W$$

In grouped convolution, input channels are divided into G subsets, with each subset connecting only to a corresponding set of convolution kernels. In this case, the computational load takes the following form:

(24) $${\mathrm{FLOPs}}_{\mathrm{gconv}}=\frac{C_{\mathrm{in}}}{G}\times C_{\mathrm{out}}\times K\times K\times H\times W$$

Which is approximately 1/G of the standard convolution. In scenarios with large channel counts in the detection head, this grouping strategy significantly reduces computational complexity while preserving necessary cross-channel interactions, thereby accelerating network training and inference speeds. Combined with multi-scale input and dual-track design, LGC-Head effectively supports real-time PCB defect detection tasks in hardware-resource-constrained industrial environments.

## 4. Experiments

### 4.1. Experimental Setup and Dataset

Experiments were conducted on a standalone workstation. The hardware platform was based on a device running the Windows 10 operating system, equipped with an NVIDIA GeForce RTX 3050 Laptop GPU as the primary computing unit, possessing 4 GB of video memory, which is sufficient to support computational tasks such as batch training and multi-scale feature inference. The software environment utilized Python 3.10.18 as the development language, with PyTorch 2.5.1+cu121 serving as the deep learning framework. The overall experimental environment is detailed in Table 1.

Key hyperparameter configurations for the model training phase are presented in Table 2. All input images were uniformly resized to 640 × 640 pixels prior to being fed into the network. Stochastic Gradient Descent (SGD) with momentum was selected as the optimizer to balance training stability and convergence speed, with the weight decay coefficient set to 5 × 10^−4^ to mitigate overfitting. The initial learning rate was set to 0.01 and the momentum coefficient to 0.937 to balance parameter update magnitudes and gradient oscillations. The batch size was set to 16, and the training duration was set to 300 epochs, enabling the model to converge stably and learn discriminative feature representations through sufficient iterations.

**Figure 5:**
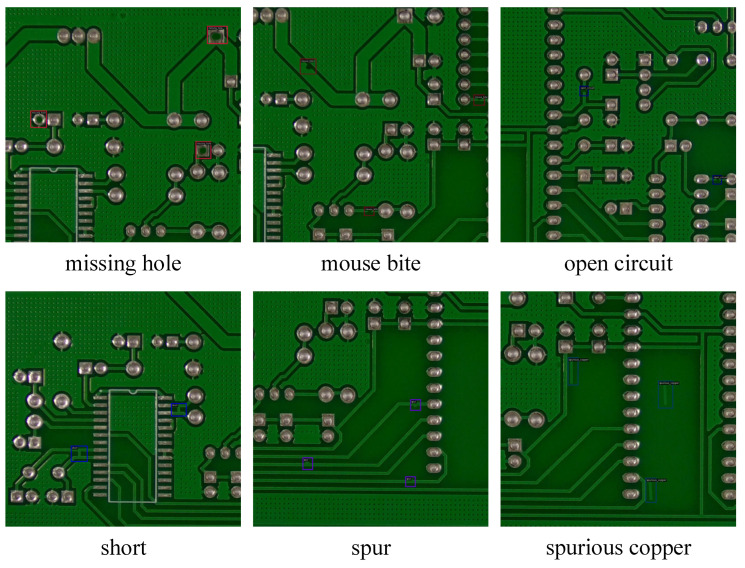
Examples of PCB defects in the dataset.

Regarding the dataset, this study selected the PKU-Market-PCB dataset [43] for experimentation. This dataset comprises PCB images acquired from actual industrial production lines, with each image annotated for defect category and location. It covers six common defect types, as illustrated in Figure 5: missing holes, mouse bites, open circuits, shorts, spurs, and spurious copper. These categories are highly consistent with common failure modes in industrial production, accurately reflecting real-world inspection scenarios.

The images were captured using a 16-megapixel high-resolution industrial camera equipped with a CMOS sensor. The system is fitted with a distortion-free, zoomable industrial lens (focal length adjustable from 6 to 12 mm, maximum aperture f/1.6) to accommodate PCBs of varying sizes and prevent edge distortion. To mitigate the adverse effects of specular reflections, shadows, and uneven illumination on subsequent analysis, two frosted ring-shaped LED light sources with specialized diffusing matte panels were employed. The original image resolution is 4608 × 3456 pixels, and during defect generation, images were resized according to the actual dimensions of each PCB to ensure data fidelity and alignment with real-world industrial applications.

Missing holes typically manifest as the complete absence of metal plating or mere residues of non-metallic voids at via locations; in images, circular or near-circular regions lose their metallic luster, and surrounding pad structures appear incomplete. These commonly occur at interlayer connection points in multi-layer boards. Mouse bites frequently appear at the edges of conductive lines, presenting as irregular, jagged indentations resembling traces left by rodent gnawing. Open circuits refer to discontinuities in copper traces that should be continuous, where conductors on either side of the break are separated; in images, this appears as a thin line abruptly interrupted. Shorts involve abnormal metallic connections between conductors that should be electrically isolated, manifesting as slender copper bridges, solder overflows, or metallic foreign object overlaps, typically exhibiting high-brightness specular reflections. Spurs are minute protrusions along conductive line edges generated by uneven etching or plating anomalies, often appearing as sharp or strip-like projections. Spurious copper refers to isolated copper foil residues in non-designed conductive areas such as solder mask layers, silk screen regions, or component gaps; their morphology is irregular, and they are easily confused with genuine traces against complex backgrounds.

**Figure 6:**
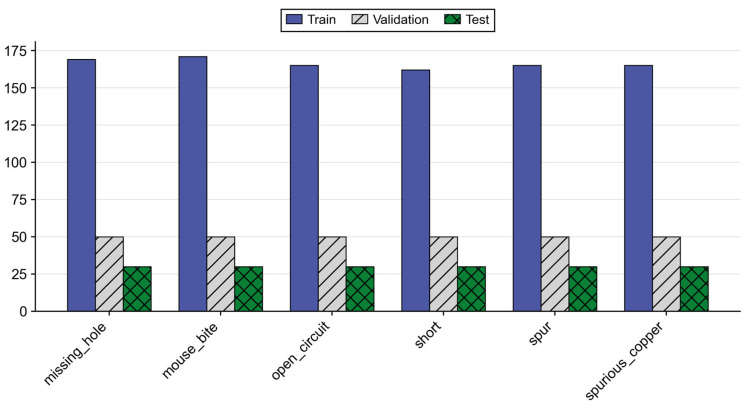
Statistical results of various defects in the dataset.

In terms of sample partition, the training set contains 169 missing hole, 171 mouse bite, 163 open-circuit, 162 short, 165 spur, and 165 spurious copper images. The validation and test sets each contain 50 and 30 samples, respectively, for each of the 6 defect categories. The overall data distribution is shown in Figure 6, ensuring a relatively balanced sample size for each defect category during both training and evaluation phases.

Model performance evaluation employs metrics including Precision, Recall, Average Precision (AP), mean Average Precision (mAP) across categories, Frames Per Second (FPS), parameter count (Parameters), and computational complexity (GFLOPs). Precision measures the proportion of true positive samples among all positive predictions made by the model, while Recall measures the proportion of true positive samples successfully detected out of all actual positive samples. The formulas for Precision and Recall are as follows:

(25) $$\mathrm{Precision}=\frac{TP}{TP+FP},\;\mathrm{Recall}=\frac{TP}{TP+FN}$$

where TP represents the number of samples correctly predicted as positive, FP represents the number of negative samples incorrectly predicted as positive, and FN represents the number of positive samples incorrectly predicted as negative.

Average Precision (AP) measures the comprehensive performance of the model under different decision thresholds by calculating the area under the Precision–Recall curve, expressed as follows:

(26) $$AP=\int_{0}^{1}p\left(r\right)dr$$

where $p\left(r\right)$ represents the Precision corresponding to a Recall rate of r. In practical calculations, discrete sampling points are typically used for numerical approximation. The arithmetic mean of APs calculated for all categories yields the mean Average Precision (mAP), expressed as follows:

(27) $$mAP=\frac{1}{N}\sum_{i=1}^{N}AP_{i}$$

where N is the number of defect categories, and $AP_{i}$ is the Average Precision for the i-th category.

Frames Per Second is defined as follows: During inference, images are fed into the model in batches of a fixed size. The average time required to process each batch, denoted as $t_{\mathrm{batch}\;}$ (in seconds), is recorded. Given a batch size of B, FPS is calculated as follows:

(28) $$\;FPS=\frac{B}{t_{\mathrm{batch}\;}}$$

This metric represents the number of images the system can process per unit time in real-world deployment; a higher FPS indicates faster inference speed and better real-time performance.

GFLOPs were computed using the thop.profile tool in the PyTorch framework by performing forward inference on the model under consistent input resolution of 640 × 640 and a batch size of 16.

Parameter count and GFLOPs reflect model scale and theoretical computational overhead, respectively; together with FPS, they are used to comprehensively assess the efficiency and resource occupancy of the model in actual industrial deployment scenarios. All models were evaluated on the same hardware platform, namely, an NVIDIA RTX 3050 Laptop GPU, using an identical software environment and testing protocol.

### 4.2. Comparative Experiments

To comprehensively validate the overall performance of MDEB-YOLO, this study conducted comparative experiments against multiple mainstream object detection models on the PKU-Market-PCB dataset. The comparative baselines encompassed two-stage detectors (Faster R-CNN, Cascade R-CNN, Libra R-CNN) and one-stage detectors (RetinaNet, CenterNet, GFL, TOOD, ATSS, RT-DETR, YOLOv5s, YOLOv8s, and YOLOv11s). The evaluation framework included Average Precision (AP) per category, mAP, parameters, FPS, and GFLOPs, aiming to assess detection accuracy, model scale, and inference efficiency from three dimensions.

The quantitative comparison results are presented in Table 3 and Table 4. On the validation set, MDEB-YOLO demonstrated significant performance advantages, achieving a mAP of 0.959, surpassing all comparative models. Compared to the strongest one-stage baseline, YOLOv11s, MDEB-YOLO improved mAP from 0.944 to 0.959 while significantly reducing parameter count from 9.42 M to 7.11 M and GFLOPs from 21.3 to 18.4. This fully demonstrates that the model achieves structural efficiency and compactness while maintaining high precision. Compared to YOLOv5s and YOLOv8s, MDEB-YOLO achieved mAP improvements of 0.024 and 0.017, respectively, with lower computational overhead. Compared to two-stage detectors such as Faster R-CNN, MDEB-YOLO not only leads significantly in accuracy but also possesses greater advantages for industrial deployment in terms of inference speed and resource utilization.

Further analysis at the category level reveals that MDEB-YOLO’s optimization effect is particularly prominent for micro-geometric defects. The data indicate decisively improved accuracy on the most challenging “Mouse bite” and “Spur” categories. Specifically, on the validation set, the AP for the mouse bite category reached 0.941, an increase of 0.040, 0.015, and 0.047 compared to YOLOv11s, YOLOv8s, and YOLOv5s, respectively; the AP for the spur category reached 0.928, an increase of 0.037, 0.017, and 0.059 compared to the three aforementioned models. Compared to other detectors such as RetinaNet and ATSS, the AP improvements for these two defect types are even more significant. These results strongly corroborate the hypothesis of this paper: the EMDA module and BRM-FPN structure effectively enhance the network’s capability to characterize and preserve fine-grained concave–convex features along conductive line edges.

On the test set, MDEB-YOLO likewise exhibited excellent generalization capabilities, achieving an overall mAP of 0.954. Compared to its closest competitor, YOLOv11s, mAP improved by 0.015, while APs for mouse bite and spur categories saw substantial increases of 0.045 and 0.046, respectively. This indicates that MDEB-YOLO not only performs excellently on specific data splits but also maintains stable robustness when encountering unseen samples.

**Figure 7:**
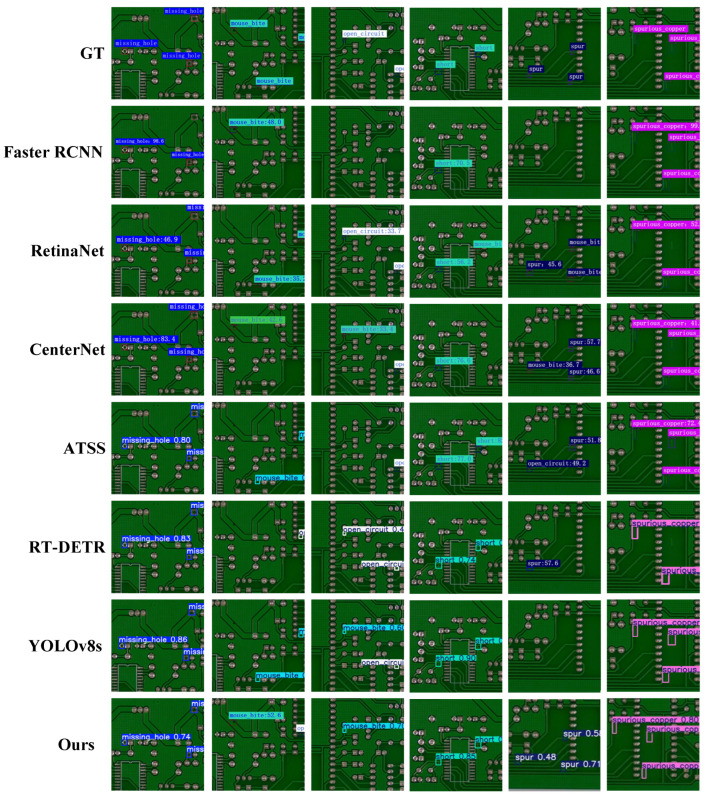
Comparison of detection results among typical models.

Figure 7 presents the visualization results of different detectors on representative samples. It can be observed that in scenarios where defect regions occupy a minimal proportion of the board area and the background texture is complex, MDEB-YOLO is capable of accurately detecting all targets and providing bounding boxes that highly conform to actual contours. In contrast, some comparative models exhibit false negatives and false positives. These visualization results are highly consistent with the quantitative data, further verifying the reliability and practical value of MDEB-YOLO in PCB micro-defect detection tasks.

### 4.3. Ablation Studies

To assess the individual contributions of the EMDA module, BRM-FPN neck network, and LGC-Head detection head to the overall model performance, this section details a stepwise ablation study. Table 5 records the AP per category, overall mAP, and GFLOPs under different module combinations.

As indicated in Table 5, the baseline model achieved a mAP of 0.944 with 21.3 GFLOPs. Introducing the EMDA module alone raised the mAP to 0.952. This suggests that enhancing the modeling of geometric contours along conductive lines within the backbone effectively improves the model’s perceptual capability for fine-grained defects. When BRM-FPN was introduced alone, mAP marginally decreased to 0.941, remaining comparable to the baseline. This implies that without upstream feature enhancement, merely increasing the complexity of feature fusion does not directly translate into accuracy gains and may even introduce feature redundancy. Adopting the LGC-Head alone unexpectedly increased mAP to 0.949 while significantly reducing GFLOPs to 19.3. This indicates that the grouped convolution design, while substantially reducing computational load, may have induced a regularization effect by reducing parameter redundancy, thereby maintaining robust representation capabilities.

When both EMDA and BRM-FPN were introduced, the combined use yielded a mAP of 0.956, clearly outperforming the use of either module in isolation. This validates the significant complementarity between backbone feature enhancement and bidirectional multi-scale fusion: EMDA provides high-quality local detail features, while BRM-FPN is responsible for effectively distributing and fusing these features across the multi-scale space. Under the full configuration with all three modules enabled, MDEB-YOLO achieved the highest mAP of 0.959, while GFLOPs dropped to a minimum of 18.4. This demonstrates that the three components form a beneficial synergistic enhancement in terms of both precision and efficiency, achieving Pareto optimality in model performance.

**Figure 8:**
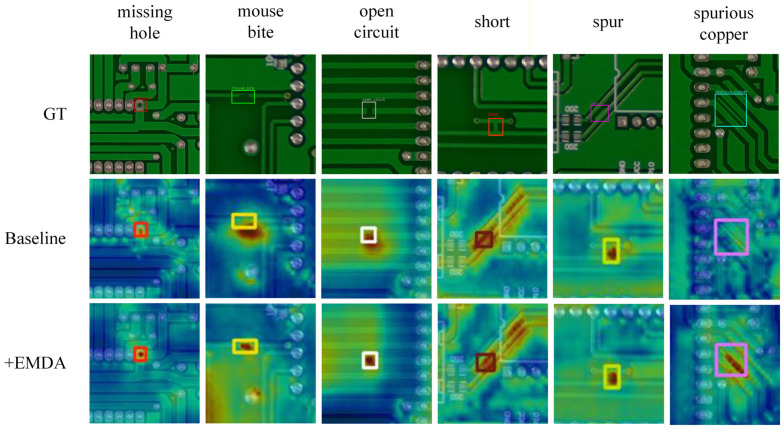
Comparison of Grad-CAM++ heatmaps between EMDA module and baseline model across six defect types.

To visually verify the effectiveness of the EMDA module, this study employs Grad-CAM++ to generate activation heatmaps for visual analysis. Grad-CAM++ utilizes pixel-level gradients to compute the importance of specific pixels to the prediction, thereby enabling the simultaneous highlighting of multiple object instances within the same image. In industrial defect detection scenarios, explainable artificial intelligence techniques are used to verify whether the model focuses on genuine defect structures [44], as shown in Figure 8. The results show that compared to the baseline model, the network with EMDA exhibits more concentrated activation responses in defect core regions, while activation in background noise regions is significantly attenuated. This confirms that EMDA effectively guides the network to focus on discriminative geometric structural features.

**Figure 9:**
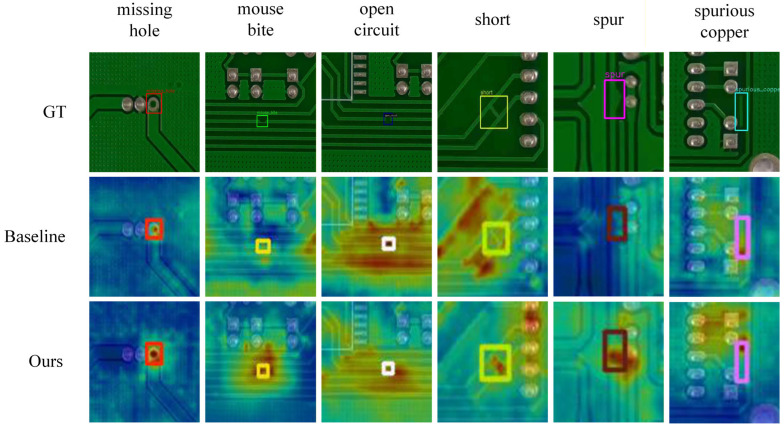
Comparison of Grad-CAM++ heatmaps between MDEB-YOLO and YOLOv11s across six defect types.

Figure 8 presents the original images, while the second and third rows show the Grad-CAM++ heatmaps generated by YOLOv11s and YOLOv11s equipped with the EMDA module, respectively. In the heatmaps, darker colors indicate stronger feature responses. Compared with the baseline, the high-activation regions exhibit a higher intersection-over-union (IoU) with the annotated defect masks, and effective defect heatmaps are observed to concentrate more than 70% of the total activation energy within the annotated defect boundaries. The introduction of EMDA further localizes the responses around defect cores and edge contours while significantly suppressing activations in non-defect background regions. This indicates that EMDA guides the network to focus more closely on concave–convex micro-notch structures along conductor edges, thereby reducing the risk of micro-defects being overwhelmed by background patterns. Building on this, to assess the overall synergistic performance of the EMDA module and the BRM-FPN neck network, further visual analysis was performed on the complete MDEB-YOLO model. Figure 9 presents a comparison of Grad-CAM++ heatmaps for YOLOv11s and MDEB-YOLO on identical samples.

As shown in Figure 9, while maintaining a fast inference speed, the activation responses of MDEB-YOLO exhibit a higher spatial consistency with the actual defect regions. From the perspective of energy distribution, the Grad-CAM++ activation energy within defect areas is significantly higher than that in the background, indicating that the model’s decision energy is more concentrated on the true defect structures. Specifically, for small and complex concave–convex defects such as mouse bites and burrs, which have limited area and intricate boundaries, traditional methods often generate irrelevant activations on surrounding circuit textures. In contrast, MDEB-YOLO demonstrates a higher defect-region energy coverage on these samples, with activation energy primarily distributed along defect edges and concave areas rather than adjacent circuit structures. This suggests that, during multi-scale feature fusion, BRM-FPN can effectively preserve and enhance the fine-grained edge responses provided by EMDA within the global context, resulting in activation energy more focused on physically real defect structures rather than background noise.

**Figure 10:**
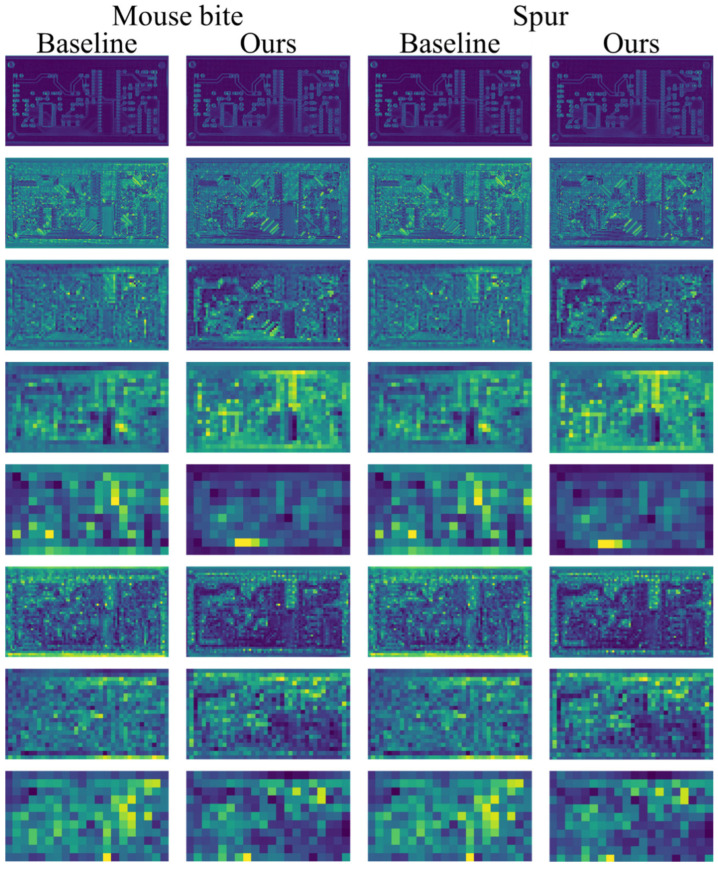
Visualization comparison of feature maps between YOLOv11s and MDEB-YOLO for mouse bite and spur defects.

To further analyze the synergy between EMDA and BRM-FPN at the feature level, this study visualized feature maps from key feature extraction and fusion layers of MDEB-YOLO for mouse bite and spur defects. Figure 10 displays the feature map comparison. For mouse bite and spur defects, the feature responses of the baseline YOLOv11s are relatively diffuse and contain substantial background noise. In contrast, MDEB-YOLO exhibits clear defect contours even in shallow feature maps and maintains compact response regions in deep feature maps. Especially for spur defects, MDEB-YOLO successfully separates the defect from the normal conductive line, fully demonstrating the synergistic advantage of EMDA and BRM-FPN in fine-grained structural modeling and multi-scale context fusion.

In summary, the ablation studies and visual analyses collectively demonstrate that the EMDA module, the BRM-FPN neck network, and the LGC-Head detection head all play pivotal roles in enhancing micro-defect detection accuracy and reducing computational complexity. Their synergistic design is the primary reason MDEB-YOLO achieves a superior balance of performance in PCB micro-defect detection tasks.

## 5. Conclusions

This study addresses the enhancement in detection accuracy and efficiency for common micro-surface defects in PCB manufacturing, proposing a novel lightweight object detection model designated as MDEB-YOLO. Targeting the difficulties inherent in traditional methods—such as challenges in extracting features from subtle concave–convex deformations along conductive line edges, severe background interference, and high computational resource consumption—this research implemented systematic innovations across three dimensions: feature modeling, multi-scale fusion, and detection head design. The experimental results indicate that the proposed model not only effectively resolves issues of missed detections and false alarms for micro-geometric defects but also achieves a significant performance balance between model lightweighting and real-time inference, offering an efficient and viable solution for online quality inspection in industrial scenarios.

Methodologically, this study first proposed the EMDA convolution module. By introducing cross-spatial channel learning and deformable attention mechanisms, it successfully achieved adaptive focusing and refined feature extraction for irregular micro-notch structures along conductive edges. Second, the designed BRM-FPN network, utilizing bidirectional residual paths and adaptive weighted fusion strategies, effectively suppressed redundant background noise and reinforced multi-scale feature expression, ensuring the complete transmission of information regarding ultra-small targets. Finally, the reconstructed LGC-Head leveraged grouped convolutions to significantly compress model parameters and computational overhead. Validation on a real-world industrial PCB dataset demonstrated that MDEB-YOLO, while maintaining a high mean Average Precision (mAP) of 95.9%, achieved an inference speed of 80.6 FPS with a parameter count of only 7.11 M. Notably, detection accuracy for spur and mouse bite defects improved by 4.0% and 3.7%, respectively, yielding comprehensive performance superior to current mainstream detection networks.

The MDEB-YOLO algorithm demonstrates strong performance in Printed Circuit Board defect detection tasks; however, several challenges remain. Under complex backgrounds—such as severe surface contamination, oxidation, or localized glare caused by highly reflective solder joints—the model’s generalization capability still requires further improvement. Moreover, industrial PCB inspection relies heavily on large-scale manually annotated datasets, leading to high labeling costs, and the current model architecture can be further lightweighted to enable efficient deployment on resource-constrained edge devices. To address these issues, future research will focus on designing more robust feature representation mechanisms by integrating domain adaptation techniques with advanced data augmentation strategies to alleviate visual interference and distribution shifts in real-world industrial scenarios, adopting lightweight backbone networks such as MobileNetV4 and EfficientNet-Lite to compress the model parameters to fewer than 5 M for efficient edge deployment, and exploring few-shot learning as well as unsupervised/self-supervised pretraining paradigms to reduce dependence on large-scale expert-annotated datasets. For example, Meta R-CNN [45] introduces class prototypes and attention mechanisms into Faster R-CNN using a meta-learning approach; FSCE [46] leverages contrastive learning to optimize candidate region features, enhancing the distinguishability of few-shot classes in the semantic space; and Hallucination FSOD [47] adopts a generative augmentation strategy to expand feature samples, thereby improving the model’s generalization ability for scarce classes, thereby enhancing the adaptability and deployment flexibility of the model across different PCB production lines and dynamically evolving manufacturing processes.

## Figures and Tables

**Table 1:** Experimental software and hardware environment.

Item	Configuration
Operating system	Windows 10
GPU	NVIDIA GeForce RTX 3050 Laptop GPU
GPU memory	4 GB
Python version	3.10.18
PyTorch version	2.5.1+cu121
Item	Configuration

**Table 2:** Hyperparameters for model training.

Hyperparameter	Value
Input size	640 × 640
Optimizer	SGD
Weight decay	5 × 10^−4^
Initial learning rate lr0	0.01
Momentum	0.937
Batch size	16
Epochs	300

**Table 3:** Accuracy of various models on the validation set.

Model	Missing Hole	Mouse Bite	Open Circuit	Short	Spur	Spurious Copper	mAP	Parameters/M	FPS	GFLOPS
RetinaNet	0.968	0.751	0.792	0.858	0.533	0.855	0.792	18.433	43.3	58.389
CenterNet	0.961	0.768	0.872	0.854	0.747	0.778	0.83	32.122	32.1	83.3
Cascade R-CNN	0.968	0.88	0.722	0.865	0.818	0.961	0.869	69.167	28.2	123
GFL	0.964	0.865	0.782	0.856	0.761	0.895	0.855	51.262	31.3	119
TOOD	0.918	0.903	0.755	0.883	0.787	0.892	0.849	53.499	34.6	83.049
Faster R-CNN	0.968	0.812	0.562	0.892	0.819	0.936	0.832	60.366	30.5	128
Libra R-CNN	0.941	0.732	0.509	0.829	0.771	0.889	0.779	60.629	32.3	130
ATSS	0.954	0.86	0.675	0.816	0.797	0.863	0.828	31.211	43.4	78.6
RT-DETR	0.988	0.933	0.961	0.851	0.828	0.84	0.900	60.91	51.91	183.6
YOLOv11s	0.995	0.901	0.958	0.96	0.891	0.959	0.944	9.42	59.17	21.3
YOLOv8s	0.995	0.926	0.94	0.936	0.911	0.944	0.942	11.12	66.7	18.7
YOLOv5s	0.985	0.894	0.952	0.975	0.869	0.938	0.935	9.2	88.49	23.8
Ours	0.995	0.941	0.962	0.968	0.928	0.965	0.959	7.11	80.65	18.4

**Table 4:** Accuracy of various models on the test set.

Model	Missing Hole	Mouse Bite	Open Circuit	Short	Spur	Spurious Copper	mAP
RetinaNet	0.954	0.743	0.781	0.885	0.526	0.855	0.79
CenterNet	0.959	0.757	0.869	0.844	0.742	0.778	0.824
Cascade R-CNN	0.947	0.862	0.732	0.859	0.817	0.942	0.86
GFL	0.952	0.857	0.779	0.852	0.76	0.889	0.848
TOOD	0.912	0.894	0.735	0.879	0.771	0.891	0.847
Faster R-CNN	0.957	0.813	0.572	0.891	0.809	0.936	0.829
Libra R-CNN	0.937	0.742	0.507	0.818	0.742	0.864	0.768
ATSS	0.943	0.852	0.669	0.804	0.781	0.862	0.818
RT-DETR	0.987	0.923	0.942	0.843	0.816	0.834	0.891
YOLOv11s	0.995	0.894	0.954	0.957	0.872	0.961	0.939
YOLOv8s	0.995	0.924	0.932	0.918	0.897	0.942	0.934
YOLOv5s	0.984	0.891	0.942	0.963	0.861	0.927	0.928
Ours	0.985	0.939	0.957	0.962	0.918	0.965	0.954

**Table 5:** Ablation study results.

EMDA	BRM-FPN	LGC-Head	Missing Hole	Mouse Bite	Open Circuit	Short	Spur	Spurious Copper	mAP	GFLOPS
			0.995	0.901	0.958	0.96	0.891	0.959	0.944	21.3
√			0.995	0.912	0.935	0.987	0.925	0.961	0.952	21.0
	√		0.995	0.914	0.94	0.973	0.866	0.96	0.941	21.6
		√	0.995	0.932	0.965	0.949	0.901	0.954	0.949	19.3
√	√		0.995	0.953	0.947	0.973	0.911	0.959	0.956	20.2
√		√	0.995	0.912	0.926	0.971	0.891	0.959	0.942	18.8
	√	√	0.995	0.907	0.951	0.949	0.875	0.963	0.940	19.0
√	√	√	0.995	0.941	0.962	0.968	0.928	0.965	0.959	18.4

## Data Availability

The original contributions presented in this study are included in the article; further inquiries regarding the PCB dataset from Peking University can be directed to the corresponding author. The PCB dataset used in this study is the PKU-Market-PCB dataset, publicly available from the Open Lab on Human–Robot Interactionof Peking University at: https://robotics.pkusz.edu.cn/resources/dataset/ (accessed on 27 January 2026).

## References

[1] Zhang Q., Liu H. Multi-scale defect detection of printed circuit board based on feature pyramid network. Proceedings of the 2021 IEEE International Conference on Artificial Intelligence and Computer Applications (ICAICA).

[2] Bajenescu T. (2021). Miniaturisation of electronic components and the problem of device overheating. Electroteh. Electron. Autom..

[3] Wu Y.Q., Zhao L.Y., Yuan Y.B., Yang J. (2022). Current status and prospect of PCB defect detection algorithm based on machine vision. Chin. J. Sci. Instrum..

[4] Mirzaei M. (2023). Automating Fault Detection and Quality Control in PCBs. Master’s Thesis.

[5] Ling Q., Isa N.A.M. (2023). Printed circuit board defect detection methods based on image processing, machine learning and deep learning: A survey. IEEE Access.

[6] Yan H., Zhang H., Gao F., Wu H., Tang S. (2024). Research on deep learning model enhancements for PCB surface defect detection. Electronics.

[7] Cheng Z., Wu Y., Li Y., Cai L., Ihnaini B. (2025). A Comprehensive Review of Explainable Artificial Intelligence (XAI) in Computer Vision. Sensors.

[8] Ren S., He K., Girshick R., Sun J. Faster R-CNN: Towards real-time object detection with region proposal networks. Proceedings of the Advances in Neural Information Processing Systems 28 (NIPS 2015).

[9] Lin T.Y., Goyal P., Girshick R., He K., Dollár P. Focal loss for dense object detection. Proceedings of the IEEE International Conference on Computer Vision (ICCV).

[10] Redmon J., Divvala S., Girshick R., Farhadi A. You only look once: Unified, real-time object detection. Proceedings of the IEEE Conference on Computer Vision and Pattern Recognition (CVPR).

[11] Zhao Y., Lv W., Xu S., Wei J., Wang G., Dang Q., Liu Y., Chen J. DETRs beat YOLOs on real-time object detection. Proceedings of the IEEE/CVF Conference on Computer Vision and Pattern Recognition (CVPR).

[12] Hoangvan X., Dinh D.B., Canh T.N., Nguyen V.-T. (2025). ESRPCB: An edge guided super-resolution model and ensemble learning for tiny printed circuit board defect detection. Eng. Appl. Artif. Intell..

[13] Pang J., Chen K., Shi J., Feng H., Ouyang W., Lin D. Libra r-cnn: Towards balanced learning for object detection. Proceedings of the IEEE/CVF Conference on Computer Vision and Pattern Recognition.

[14] Guo C., Fan B., Zhang Q., Xiang S., Pan C. Augfpn: Improving multi-scale feature learning for object detection. Proceedings of the IEEE/CVF conference on COMPUTER Vision and Pattern Recognition.

[15] Wang J., Chen K., Xu R., Liu Z., Loy C.C., Lin D. Carafe: Content-aware reassembly of features. Proceedings of the IEEE/CVF International Conference on Computer Vision.

[16] Bhattacharya A., Cloutier S.G. (2022). End-to-end deep learning framework for printed circuit board manufacturing defect classification. Sci. Rep..

[17] Yu X., Li H.X., Yang H. (2023). Collaborative learning classification model for PCBs defect detection against image and label uncertainty. IEEE Trans. Instrum. Meas..

[18] Chen B., Dang Z. (2023). Fast PCB defect detection method based on FasterNet backbone network and CBAM attention mechanism integrated with feature fusion module in improved YOLOv7. IEEE Access.

[19] Liao H.C., Lim Z.Y., Hu Y.X., Tseng H.-W. Guidelines of automated optical inspection (AOI) system development. Proceedings of the 2018 IEEE 3rd International Conference on Signal and Image Processing (ICSIP).

[20] Yu W. (2007). Research on Application of AOI Technology in PCB Defect Detection. Doctoral Dissertation.

[21] Tao X., Hou W., Xu D. (2021). A survey of surface defect detection methods based on deep learning. Acta Autom. Sin..

[22] Yun T.S., Sim K.J., Kim H.J. (2000). Support vector machine-based inspection of solder joints using circular illumination. Electron. Lett..

[23] Belbachir A.N., Lera M., Fanni A., Montisci A. An automatic optical inspection system for the diagnosis of printed circuits based on neural networks. Proceedings of the IEEE Industry Applications Society Annual Meeting.

[24] Wang J.Q., Chen K., Yang S., Loy C.C., Lin D. Region proposal by guided anchoring. Proceedings of the IEEE/CVF Conference on Computer Vision and Pattern Recognition (CVPR).

[25] Liu Y.Q., Li W., Li Y.C. Network traffic classification using k-means clustering. Proceedings of the Second International Multi-Symposiums on Computer and Computational Sciences.

[26] Hu B., Wang J. (2020). Detection of PCB surface defects with improved Faster-RCNN and feature pyramid network. IEEE Access.

[27] Wu H., Gao W., Xu X. (2019). Solder joint recognition using mask R-CNN method. IEEE Trans. Compon. Packag. Manuf. Technol..

[28] Howard A.G., Zhu M., Chen B., Kalenichenko D., Wang W., Weyand T., Andreetto M., Adam H. (2017). Mobilenets: Efficient convolutional neural networks for mobile vision applications. arXiv.

[29] Sandler M., Howard A., Zhu M., Zhmoginov A., Chen L. MobileNetV2, Inverted residuals and linear bottlenecks. Proceedings of the IEEE Conference on Computer Vision and Pattern Recognition (CVPR).

[30] Howard A., Sandler M., Chen B., Wang W., Chen L.-C., Tan M., Chu G., Vasudevan V., Zhu Y., Pang R. Searching for MobileNetV3. Proceedings of the IEEE International Conference on Computer Vision (ICCV).

[31] Han K., Wang Y., Tian Q., Guo J., Xu C., Xu C. GhostNet: More features from cheap operations. Proceedings of the IEEE/CVF Conference on Computer Vision and Pattern Recognition (CVPR).

[32] Zhang X., Zhou X., Lin M., Sun J. ShuffleNet: An extremely efficient convolutional neural network for mobile devices. Proceedings of the IEEE Conference on Computer Vision and Pattern Recognition (CVPR).

[33] Ma N., Zhang X., Zheng H.T., Sun J. ShuffleNet V2, Practical guidelines for efficient CNN architecture design. Proceedings of the European Conference on Computer Vision (ECCV).

[34] Tan M., Le Q.V. (2019). EfficientNet: Rethinking model scaling for convolutional neural networks. Proceedings of the 36th International Conference on Machine Learning (ICML).

[35] An K., Zhang Y. (2022). LPViT: A transformer based model for PCB image classification and defect detection. IEEE Access.

[36] Liu T., Cao G.Z., He Z., Xie S. (2023). Refined defect detector with deformable transformer and pyramid feature fusion for PCB detection. IEEE Trans. Instrum. Meas..

[37] Hou Q., Zhou D., Feng J. Coordinate attention for efficient mobile network design. Proceedings of the IEEE/CVF Conference on Computer Vision and Pattern Recognition.

[38] Selvaraju R.R., Cogswell M., Das A., Vedantam R., Parikh D., Batra D. Grad-cam: Visual explanations from deep networks via gradient-based localization. Proceedings of the IEEE International Conference on Computer Vision.

[39] Petsiuk V., Das A., Saenko K. (2018). Rise: Randomized input sampling for explanation of black-box models. arXiv.

[40] Chefer H., Gur S., Wolf L. Transformer interpretability beyond attention visualization. Proceedings of the IEEE/CVF Conference on Computer Vision and Pattern Recognition.

[41] Huang Z., Yao X., Liu Y., Dumitru C.O., Datcu M., Han J. (2022). Physically explainable CNN for SAR image classification. ISPRS J. Photogramm. Remote Sens..

[42] Cuomo S., Di Cola V.S., Giampaolo F., Rozza G., Raissi M., Piccialli F. (2022). Scientific machine learning through physics–informed neural networks: Where we are and what’s next. J. Sci. Comput..

[43] Huang W., Wei P., Zhang M., Liu H. (2020). HRIPCB: A challenging dataset for PCB defects detection and classification. J. Eng..

[44] Chattopadhay A., Sarkar A., Howlader P., Balasubramanian V.N. Grad-cam++: Generalized gradient-based visual explanations for deep convolutional networks. Proceedings of the 2018 IEEE Winter Conference on Applications of Computer Vision (WACV).

[45] Yan X., Chen Z., Xu A., Wang X., Liang X., Lin L. Meta r-cnn: Towards general solver for instance-level low-shot learning. Proceedings of the IEEE/CVF International Conference on Computer Vision.

[46] Sun B., Li B., Cai S., Yuan Y., Zhang C. Fsce: Few-shot object detection via contrastive proposal encoding. Proceedings of the IEEE/CVF Conference on Computer Vision and Pattern Recognition.

[47] Zhang W., Wang Y.X. Hallucination improves few-shot object detection. Proceedings of the IEEE/CVF Conference on Computer Vision and Pattern Recognition.

